# Learning to see the invisible: A data‐driven approach to finding the underlying patterns of abnormality in visually normal brain magnetic resonance images in patients with temporal lobe epilepsy

**DOI:** 10.1111/epi.16380

**Published:** 2019-11-06

**Authors:** Oscar F. Bennett, Baris Kanber, Chandrashekar Hoskote, M. Jorge Cardoso, Sebastien Ourselin, John S. Duncan, Gavin P. Winston

**Affiliations:** ^1^ Centre for Medical Image Computing Department of Medical Physics and Biomedical Engineering University College London London UK; ^2^ Department of Clinical and Experimental Epilepsy Queen Square Institute of Neurology University College London London UK; ^3^ MRI Unit Epilepsy Society Chalfont St Peter UK; ^4^ Lysholm Department of Neuroradiology National Hospital for Neurology and Neurosurgery London UK; ^5^ School of Biomedical Engineering & Imaging Sciences King’s College London London UK; ^6^ Department of Medicine Division of Neurology Queen's University Kingston Ontario Canada

**Keywords:** abnormality, data‐driven, epilepsy, machine learning, MRI‐negative

## Abstract

**Objective:**

To find the covert patterns of abnormality in patients with unilateral temporal lobe epilepsy (TLE) and visually normal brain magnetic resonance images (MRI‐negative), comparing them to those with visible abnormalities (MRI‐positive).

**Methods:**

We used multimodal brain MRI from patients with unilateral TLE and employed contemporary machine learning methods to predict the known laterality of seizure onset in 104 subjects (82 MRI‐positive, 22 MRI‐negative). A visualization approach entitled "Importance Maps" was developed to highlight image features predictive of seizure laterality in both the MRI‐positive and MRI‐negative cases.

**Results:**

Seizure laterality could be predicted with an area under the receiver operating characteristic curve of 0.981 (95% confidence interval [CI] =0.974‐0.989) in MRI‐positive and 0.842 (95% CI = 0.736‐0.949) in MRI‐negative cases. The known image features arising from the hippocampus were the leading predictors of seizure laterality in the MRI‐positive cases, whereas widespread temporal lobe abnormalities were revealed in the MRI‐negative cases.

**Significance:**

Covert abnormalities not discerned on visual reading were detected in MRI‐negative TLE, with a spatial pattern involving the whole temporal lobe, rather than just the hippocampus. This suggests that MRI‐negative TLE may be associated with subtle but widespread temporal lobe abnormalities. These abnormalities merit close inspection and postacquisition processing if there is no overt lesion.

**Key Points**

- This study was conducted to determine the covert patterns of abnormality in patients with unilateral TLE and visually normal brain MR images
- Multimodal brain MRI and contemporary machine learning methods were used, and a visualization approach entitled "Importance Maps" was developed
- Covert abnormalities in MRI‐negative TLE had a spatial pattern involving the whole temporal lobe, rather than just the hippocampus, and assisted lateralization

## 1 INTRODUCTION

Epilepsy is a common and serious neurological disease that can have a devastating effect on a person's life if not adequately treated. However, one‐third of patients continue to have frequent seizures despite optimal medical therapy.^1^, ^2^ Focal epilepsy accounts for a significant proportion (around two‐thirds) of those cases that do not respond to medication, and the temporal lobe is the most common site of onset of focal seizures. If focal epilepsy does not respond to medication, neurosurgical procedures can be considered. If the underlying epileptogenic abnormality can be located in a patient's brain and surgically removed, there is a good chance that a patient can have freedom from seizures postoperatively; up to a 82% chance of postoperative remission for at least 1 year and 41% sustained complete seizure freedom have been demonstrated.^3^

The identification of an underlying abnormality on magnetic resonance imaging (MRI) is a key goal of the surgical workup, but such abnormalities are only demonstrated on conventional MRI in about two‐thirds of cases (the so‐called MRI‐positive cases).^2^ This leaves one‐third of pharmacoresistant patients with no visually identifiable target for neurosurgery (the MRI‐negative cases). This MRI‐negative subgroup of patients represents a significant clinical challenge and is far less likely to experience seizure freedom after surgery.^3^, ^4^ In these patients, further investigations are necessary to localize the epileptogenic focus. This often involves intracranial electroencephalographic (EEG) monitoring, which carries a significant risk to the patient as well as considerable cost. It would be very beneficial to develop means of localizing underlying cerebral abnormalities using noninvasive methods such as MRI and scalp EEG alone.

Detection of brain abnormalities on magnetic resonance (MR) images using contemporary methods such as machine learning is an emerging and promising area of research.^5^, ^6^, ^7^, ^8^, ^9^, ^10^ Focke et al generated maps of gray matter voxel relevance across the brain by visualizing the weights assigned to each feature by a support vector machine (SVM) classifier trained to lateralize temporal lobe epilepsy (TLE) in 38 MRI‐positive patients.^6^ These relevance maps provided an interesting demonstration of the distribution of morphological gray matter changes found in MRI‐positive TLE patients.

Keihaninejad et al reported a distribution of brain abnormalities using an SVM classifier trained to lateralize TLE, and also included MRI‐negative cases.^7^ However, only regional brain volumes were used in the analysis, and no other relevant imaging parameters were included such as T1 or fluid‐attenuated inversion recovery (FLAIR) signal intensity. Keller and Roberts reviewed voxel‐based morphometry approaches to reporting the patterns of abnormality associated with TLE, limiting the assessment to the measurement of brain volume changes.^8^ Cantor‐Rivera et al studied correlation and analysis of variance–based feature selection methods and presented a collection of anatomical regions of interest that were found to be relevant for the purpose of TLE lateralization,^9^ but did not compare MRI‐positive and MRI‐negative cases.

Several different approaches have shown promise in lateralizing seizure origin in TLE, although mostly in MRI‐positive cases. In the case of MRI‐negative TLE, if a trained classifier is demonstrating reasonable predictive capability, then these apparently normal images must contain abnormalities, albeit subtle or poorly understood ones. Determining these abnormalities would provide valuable insight into why visual inspection might fail in the MRI‐negative cases and may guide more principled and accurate visual image interpretation in the future.

In this study, we trained a random forest classifier (RFC) on image features from multimodal MRI (T1, T2, and FLAIR) and carried out feature importance measurements, demonstrating the patterns of abnormality across the temporal lobe in both MRI‐positive and MRI‐negative cases. We created visualizations of these results, which we call "Importance Maps," that show the spatial pattern of abnormalities across the temporal lobes in these two patient groups.

## 2 MATERIALS AND METHODS

The study cohort included patients diagnosed with TLE by a consultant epileptologist. The patients underwent routine clinical scans including T1, T2, and FLAIR volumes on a 3T Signa HDx scanner (General Electric Company) at the Epilepsy Society MRI Unit, Chalfont St Peter between 2009 and 2013. Patients were included if they had provided written, informed consent and clinical consensus at the multidisciplinary epilepsy surgery meeting or careful review of notes and investigations (OFB, GPW) demonstrated findings consistent with unilateral TLE. The study was approved by the National Hospital for Neurology and Neurosurgery and the Queen Square Institute of Neurology Joint Research Ethics Committee. Starting from a dataset of 135 cases, image volumes were visually inspected and reports from a consultant neuroradiologist were reviewed to exclude cases that met any of the following criteria:

1. Previous neurosurgical excision of brain tissue (13 excluded)
2. Brain images with one or more visually apparent space‐occupying lesion (tumors, cavernomas, or arteriovenous malformations; 18 excluded)

This left 104 cases for use in the analysis: 82 MRI‐positive and 22 MRI‐negative. In all cases, the seizure laterality was established through the combination of all relevant information, including clinical semiology, radiological findings on MRI, positron emission tomography, and/or ictal single photon emission computed tomography, scalp video EEG, and where available intracranial EEG and/or magnetoencephalography, neuropsychology, and postoperative histology of resected tissues. The proportion of left‐ and right‐lateralized cases was approximately equal for the MRI‐positive and the MRI‐negative patient groups (left/right: 43/39 MRI‐positive; 10/12 MRI‐negative).

Patient demographics and a clinical summary are shown in Table 1, and detailed clinical information for the MRI‐negative cases is given in Table S1.

**Table 1:** Patient demographics and clinical summary

	MRI‐positive	MRI‐negative
Patients, n	82	22
Age^a^	39.5 (18‐63)	31.7 (19‐49)
Gender, n, male (female)	30 (52)	9 (13)
Age at disease onset^a^	13.9 (0.3‐41)	20.1 (4‐47)
Disease duration^a^	25.8 (3‐54)	11.6 (2‐32)
Had surgery and histology, n	55/82	10/22
ILAE outcomes at 12 months after surgery, n^b^		
1, seizure‐free	34	5
2	8	1
3	5	0
4‐5	5	3

Abbreviations: ILAE, International League Against Epilepsy; MRI, magnetic resonance imaging.

a Given in years as mean (range).

b These data were not available for 3 MRI‐positive patients and 1 MRI‐negative patient.

For the MRI‐positive group, 68 of 82 patients had imaging findings consistent with hippocampal sclerosis, whereas 5 of 82 patients had findings consistent with focal cortical dysplasia. The remaining 9 of 82 patients had a visually recognizable abnormality within the temporal lobe that allowed lateralization of the seizure focus but without a confident underlying diagnosis of the lesion from the radiologist.

The numbers of patients in the MRI‐positive and MRI‐negative groups who had surgery and histology were 55 of 82 and 10 of 22, respectively. International League Against Epilepsy outcomes at 12 months after surgery are shown in Table 1. In the MRI‐positive group, 34 of the 55 patients who had surgery were seizure‐free at 12 months, whereas this number was 5 of 10 for the MRI‐negative patients.

### 2.1 Workflow

**Figure 1:**
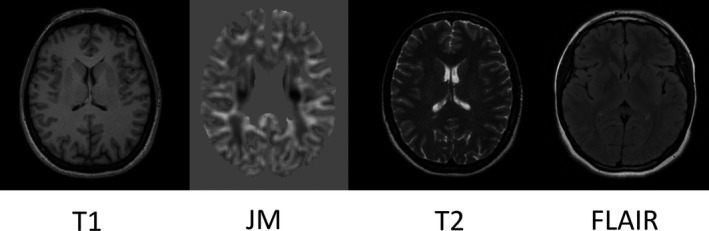
Examples of the magnetic resonance (MR) or MR‐derived image volumes used in the study: a T1‐weighted image volume, a junction map (JM),^13^ a T2‐weighted image volume, and a fluid‐attenuated inversion recovery (FLAIR) volume

Our analysis used T1‐weighted, T2‐weighted, and FLAIR MR image volumes. The T2‐weighted and FLAIR volumes were rigidly registered to the T1, and each T1‐weighted image volume was parcellated into its constituent anatomical regions using the Geodesic Information Flows (GIF) method.^11^ A junction map (JM) was also generated from each T1‐weighted volume using SPM12^12^ and locally developed MATLAB (MathWorks) scripts. This allowed us to incorporate into the analysis regions of gray‐white matter boundary blurring, an image feature often seen in focal cortical dysplasia.^13^, ^14^ Example volumes are shown in Figure 1.

The following measurements were then made for regions of the temporal lobe parcellated by GIF:

1. Right/left ratios between the volumes of each of the regions within the temporal lobes (13 features in total).
2. Right/left ratios between the means of the image intensities within each temporal lobe region for the T1, T2, FLAIR, and JM image volumes (52 features in total).
3. Right/left ratios between the standard deviations of the image intensities within each temporal lobe region for the T1, T2, FLAIR, and JM image volumes (52 features in total).

This provided a set of 117 features per patient.

These features were used to train an RFC^15^ comprising an ensemble of 5000 decision trees to predict the lateralization of the seizure origin. The importance of each of the 117 features described above for determining the seizure laterality was evaluated by calculating the reduction in Gini impurity provided by a split in that feature (weighted by the chance of reaching that node) averaged across all trees in the ensemble. Put simply, this measured the degree to which each image feature was able to separate the two classes (left TLE and right TLE) in the dataset. Once these importance measurements were calculated, visualizations of the results were generated.

To ensure the validity of the feature importances determined above, we evaluated the predictive capability of these features using SVMs. The type of kernel used for the MRI‐positive cases was linear, whereas for the MRI‐negative cases the radial basis function was chosen due to laterality detection in MRI‐negative cases being a significantly more difficult problem. We used a different type of classifier (SVM) from that used for determining the feature importances (RFC) to ensure the generalizability of the results. Hyperparameters of the SVM classifier were optimized using the grid‐search method, and its capability to lateralize the seizure origin was assessed using bolstering,^16^ a technique that provides an unbiased estimation of a classifier's performance by carrying out multiple train/test runs using multiple random partitions of the dataset into training and testing sets. We carried out 100 train/test runs, and the dataset was randomly partitioned into four folds during each run (three folds used for training, and the remaining fold used for testing). A binomial distribution was assumed on the collection of performance figures across subjects, and then the Agresti‐Coull method of interval estimation^17^ was used to produce confidence intervals (CIs).

## 3 RESULTS

**Figure 2:**
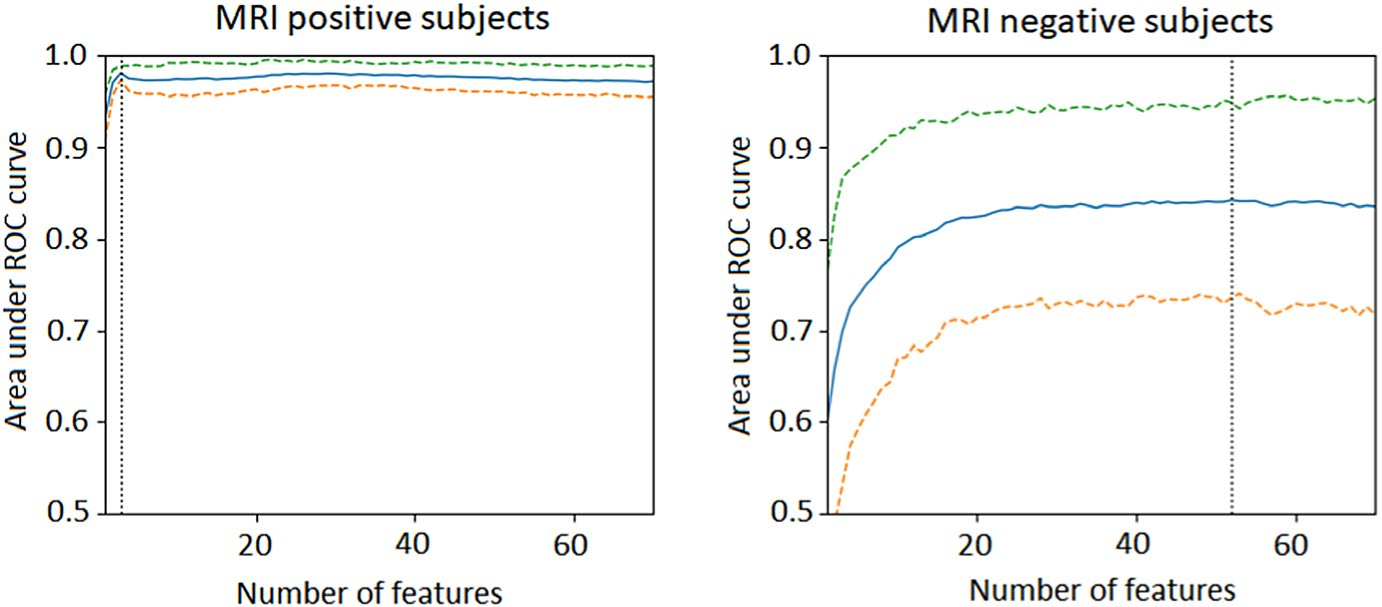
Area under the receiver operating characteristic (ROC) curve measurements obtained using the top n most important features (for n = 1‐70) in the magnetic resonance imaging (MRI)‐positive (left), and MRI‐negative cases (right). The solid blue lines represent the mean area under the ROC curve obtained in Bolstering runs and the dashed lines the edges of the 95% confidence intervals. Maximum lateralization areas under the ROC curve in the MRI‐positive and MRI‐negative cases (indicated on the plots by the vertical dotted black lines) were obtained using the top three and 52, features respectively

**Figure 3:**
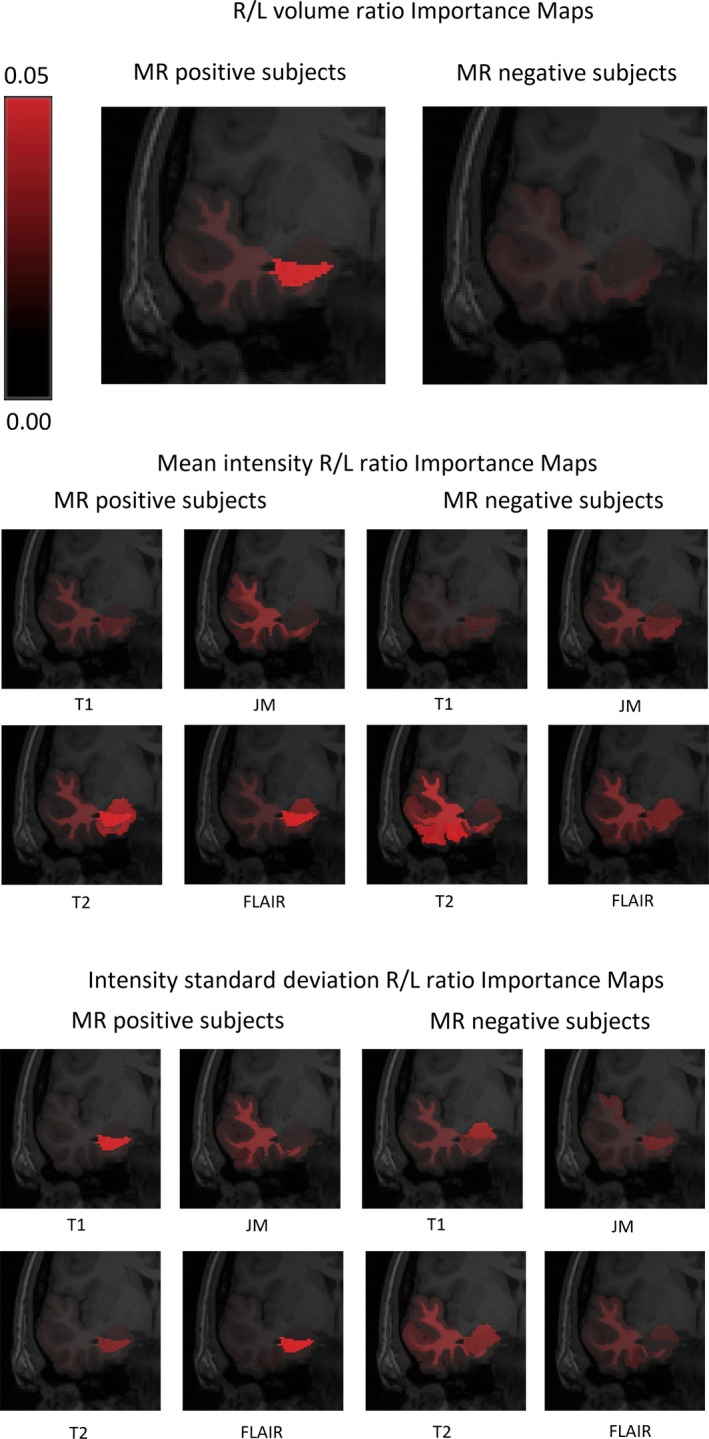
Importance Maps for regional volume asymmetry (top), mean intensity asymmetry (middle), and intensity standard deviation asymmetry (bottom) features in each image type across the temporal lobe for magnetic resonance imaging (MRI)‐positive (left) and MRI‐negative cases (right). The intensity of red within the maps signifies the importance assigned to each region. FLAIR, fluid‐attenuated inversion recovery; JM, junction map; L, left; MR, magnetic resonance; R, right

The performance of the SVM classifier at lateralizing the seizure origin was as follows: a maximum area under the receiver operating characteristic (ROC) curve of 0.981 (95% CI = 0.974‐0.989) was achieved in the MRI‐positive and 0.842 (95% CI = 0.736‐0.949) in the MRI‐negative cases (Figure 2). The corresponding classification accuracies (the agreement between the laterality determined using our method and the clinical lateralization) were 0.926 (95% CI = 0.900‐0.951) and 0.756 (95% CI = 0.636‐0.877), respectively. The most important features for predicting seizure laterality in the MRI‐positive and MRI‐negative cases are as shown in Tables 2 and 3, and visualization of these features is displayed in Figure 3.

**Table 2:** The most important image features predictive of seizure laterality in the MRI‐positive cases

Rank	Region	Feature	Gini importance
1	Hippocampus	vol	0.093198
2	Hippocampus	mean T2	0.091338
3	Hippocampus	std FLAIR	0.086794

Note

Features are vol for left/right volume ratios, mean T2 for the left/right mean T2 signal ratios, and std FLAIR for the left/right FLAIR signal standard deviation ratios. The best predictions for the MRI‐positive cases were obtained using these three features.

Abbreviations: FLAIR, fluid‐attenuated inversion recovery; MRI, magnetic resonance imaging.

**Table 3:** The most important image features predictive of seizure laterality in the MRI‐negative cases

Rank	Region	Feature	Gini importance
1	Fusiform gyrus	mean T2	0.055023
2	Inferior temporal gyrus	mean T2	0.043804
3	Temporal white matter	mean T2	0.038153
4	Amygdala	std T1	0.034179
5	Entorhinal area	std JM	0.028027
6	Amygdala	std T2	0.026306
7	Temporal white matter	std T2	0.024954
8	Hippocampus	std T2	0.024148
9	Parahippocampal gyrus	mean JM	0.022997
10	Hippocampus	mean FLAIR	0.022277
11	Temporal white matter	mean FLAIR	0.021808
12	Hippocampus	std JM	0.021753
13	Amygdala	mean FLAIR	0.021109
14	Middle temporal gyrus	mean T2	0.018927
15	Temporal white matter	mean JM	0.018077
16	Hippocampus	std T1	0.017328
17	Parahippocampal gyrus	mean T2	0.017214
18	Temporal white matter	std T1	0.016872
19	Hippocampus	mean JM	0.01663
20	Planum temporale	mean T2	0.01607
21	Temporal white matter	std FLAIR	0.014911
22	Planum polare	std JM	0.014251
23	Middle temporal gyrus	std T2	0.013715
24	Fusiform gyrus	std FLAIR	0.012645
25	Planum temporale	mean JM	0.012359
26	Hippocampus	mean T1	0.012351
27	Transverse temporal gyrus	mean T2	0.011353
28	Fusiform gyrus	mean FLAIR	0.011241
29	Planum temporale	std T2	0.011213
30	Entorhinal area	mean FLAIR	0.010305
31	Amygdala	std FLAIR	0.010257
32	Amygdala	mean T2	0.009462
33	Transverse temporal gyrus	mean JM	0.009348
34	Planum temporale	mean FLAIR	0.00865
35	Parahippocampal gyrus	vol	0.008582
36	Superior temporal gyrus	mean T2	0.008059
37	Planum polare	std T2	0.007733
38	Planum polare	mean T2	0.00762
39	Inferior temporal gyrus	mean FLAIR	0.007532
40	Amygdala	std JM	0.006893
41	Inferior temporal gyrus	std T2	0.006681
42	Parahippocampal gyrus	mean T1	0.006672
43	Planum polare	std FLAIR	0.006664
44	Transverse temporal gyrus	mean T1	0.006535
45	Inferior temporal gyrus	mean JM	0.006363
46	Entorhinal area	vol	0.006335
47	Superior temporal gyrus	std T2	0.005849
48	Planum temporale	std T1	0.005751
49	Temporal pole	mean JM	0.005662
50	Superior temporal gyrus	mean FLAIR	0.005349
51	Parahippocampal Gyrus	std JM	0.005273
52	Planum polare	mean JM	0.005273

Note

Features are vol for left/right volume ratios, mean (T1/T2/FLAIR/JM) for the left/right mean (T1/T2/FLAIR/JM) signal ratios, and std (T1/T2/FLAIR/JM) for the left/right (T1/T2/FLAIR/JM) signal standard deviation ratios. The best predictions for the MRI‐negative cases were obtained using these 52 features.

Abbreviations: FLAIR, fluid‐attenuated inversion recovery; JM, junction map; MRI, magnetic resonance imaging.

Four observations were made. First, hippocampal volume changes seen in MRI‐positive cases were essentially absent in the MRI‐negative group. Second, the typical hippocampal signal abnormalities seen in the MRI‐positive cases, and which are often focused on by radiologists performing this task visually,^14^ were less important for predicting seizure laterality in the MRI‐negative cases. Third, signal abnormalities within the amygdala, and signal abnormalities arising from within the white matter of the temporal lobe, along with inferior regions of the cortex provided useful information in the MRI‐negative group. Last, JMs, although not informative for the MRI‐positive cases, were helpful in predicting seizure laterality in MRI‐negative patients.

A re‐review of the MRI‐negative images, guided by a neuroradiologist, revealed subtle signal abnormalities in 8 of the 22 cases (Table 3), supporting our findings (Table S2 and Figure S1).

## 4 DISCUSSION

The results of our study support the hypothesis that subtle abnormalities exist in MR scans in MRI‐negative TLE, which may be detectable using contemporary machine learning approaches. The areas under the ROC curve of 0.981 (95% CI = 0.974‐0.989) and 0.842 (95% CI = 0.736‐0.949) obtained in determining seizure laterality in MRI‐positive and MRI‐negative cases, respectively, are similar to those reported in other studies addressing TLE lateralization.^7^, ^9^ However, a direct comparison cannot be made due to differences in the study cohorts.

Maximum areas under the ROC curve for lateralization in the MRI‐positive and MRI‐negative cases were obtained using the top three and 52 imaging features, respectively (Figure 2). The higher number of imaging features required for lateralization of the MRI‐negative cases is indicative of more diffuse, and perhaps more complex, imaging abnormalities in MRI‐negative epilepsy.

The abnormality patterns demonstrated in the Importance Maps are the most significant results in our study. A comparison between them suggests that there is a fundamental difference in the pattern of imaging abnormalities within the MRI‐negative cases compared to the visually recognizable patterns in the MRI‐positive cases. In other words, the MRI‐negative abnormalities are not simply a more subtle version of those seen in MRI‐positive patients.

This observed difference is in accord with other investigations. For example, MRI‐negative TLE subjects have been demonstrated to exhibit significantly different connectivity patterns from those of MRI‐positive TLE subjects,^18^ suggesting a underlying difference in the pathophysiology between the two groups.

A prominent difference in our study was the contrasting significance of the hippocampus and the other temporal lobe regions in the MRI‐positive and MRI‐negative cases, respectively. The hippocampus is conventionally focused on by radiologists reporting MRI scans in patients with TLE, as hippocampal sclerosis is a common cause of TLE.^2^ Hippocampal sclerosis is generally visually recognized from a reduced hippocampal volume and a higher T2/FLAIR signal arising from within the hippocampus.^14^ It is of note that these features top the list of important features in the MRI‐positive cases (Table 2). However, these features appear to be less important in MRI‐negative cases. Instead, multiple imaging features arising from widespread temporal lobe areas seem to be much more important in predicting seizure laterality in MRI‐negative TLE. Of note are the signal abnormalities arising from the amygdala, the temporal white matter, and the inferior regions of the cortex (Table 3, Figure 3). Our results suggest that lateralization of MRI‐negative cases in clinical practice may require a comparison of the signal intensities (“mean” features), and signal heterogeneities (“standard deviation” features) between the two brain hemispheres for widespread regions of the temporal lobe, in particular the amygdala, the temporal white matter, and the inferior regions of the cortex.

Our results provide a novel insight into why human readers may not visually identify the patterns of abnormality in certain images. The results suggest that, in cases that are difficult to lateralize using the conventional approach, it would be useful to carefully examine images for possible abnormalities and asymmetry within the amygdala, the temporal white matter, and inferior cortical regions of the temporal lobe. Postacquisition processing of these regions in such cases may also prove useful and help to unveil the subtle changes that elude visual inspection.

There are limitations to our approach. First, it is not possible to compare the pattern of detected image abnormalities with the histological ground truth in every case, because surgery is not always performed. Second, the use of simple imaging features such as regional volumes and signal variations remain fairly basic when compared to the myriad of more sophisticated image features that are available in the field of medical image computing.^19^, ^20^ However, this is also an advantage, as the use of basic image features results in Importance Maps that are more readily interpretable. More complicated features could be used, but a balance between complexity and interpretability needs to be struck. It is our aspiration that our results could be used by radiologists (perhaps aided by some simple postacquisition processing) to guide the interpretation of MRI‐negative images, and this sort of translation would only be possible with fairly simple image features like the ones used in this study.

A radiological re‐review of the MRI‐negative cases in our study already revealed subtle signal abnormalities aiding lateralization in 8 of the 22 cases. We expect further work in this area, particularly appropriate postprocessing methods enabled by machine learning, could help highlight signal abnormalities, and enable higher rates of successful radiological assessment.

## 5 CONCLUSION

This study has demonstrated that covert abnormalities exist in multimodal MR scans in MRI‐negative TLE, and that these abnormalities follow a different pattern from those in MRI‐positive TLE. These findings could aid clinical reading of MRI‐negative images in the future. The study also introduced the concept of Importance Maps, which may provide an elegant and principled way to characterize patterns of image abnormality that have previously eluded visual detection. They could be used by radiologists to help guide image evaluation in these more challenging cases.

## CONFLICT OF INTEREST

None of the authors has any conflict of interest to disclose. We confirm that we have read the Journal's position on issues involved in ethical publication and affirm that this report is consistent with those guidelines.
