# Supplementary material for: Learning to see the invisible: A data‐driven approach to finding the underlying patterns of abnormality in visually normal brain magnetic resonance images in patients with temporal lobe epilepsy
Source: Epilepsia. 2019 Nov 6;60(12):2499–507. doi: 10.1111/epi.16380 (PMC6972547; doi:10.1111/epi.16380)
Supplement: Supplementary file 1 [file EPI-60-2499-s001.docx]

**Supplementary Table 1 –** Detailed clinical information for the MRI negative cases.

| **Case** | **Semiology** | **EEG** | **EEG Findings Preop** | **Psychology** | **PET** | **Presumed seizure onset zone** | **Outcome** |
| --- | --- | --- | --- | --- | --- | --- | --- |
| 1 | Temporal, not lateralised | Left | Mid-posterior temporal (interictal, ictal) | Left temporal | Left temporo-parietal | Left fusiform gyrus | No surgery (language risk) |
| 2 | Left temporal | Left | Temporal (interictal, ictal) | Not lateralising/localising | Left temporo-parietal | Left temporal neocortex | No surgery (patient declined icEEG) |
| 3 | Temporal, not lateralised | Left | Anterior inferior temporal (ictal), bitemporal L>R (interictal) | Left frontotemporal | Left temporal | Left basal temporal | No surgery (icEEG not conclusive) |
| 4 | Temporal, not lateralised | Left | Anterior-mid temporal (interictal, ictal) | Left hemisphere, not localised | Normal | Left posterior STG | Lesionectomy STG/MTG - gliosis |
| 5 | Temporal, not lateralised | Left | Temporal (interictal, ictal) | Not lateralising/localising | Left temporal | Left temporal, likely neocortical | No surgery (patient declined icEEG) |
| 6 | Temporal, not lateralised | N/A | Normal | Left temporal | Left temporal | Left temporal (non-localised) | No surgery (not considered) |
| 7 | Left temporal | Left | Temporal (ictal) | Not lateralising/localising | Left frontal | Left temporal (non-localised) | No surgery (patient declined icEEG) |
| 8 | Temporal, not lateralised | Left | Temporal (ictal) | Left frontotemporal | Normal | Left mesial temporal | Pathology pending |
| 9 | Temporal, not lateralised | Left | Posterior temporal (interictal) | Not lateralising/localising | N/A | Left posterior temporal | No surgery (not considered) |
| 10 | Left temporal | Left | Posterior temporal (interictal) | Left temporal | N/A | Left posterior temporal | No surgery (not considered) |
| 11 | Temporal, not lateralised | Right | Posterior temporo-parietal (interictal, ictal) | Left temporal (spread) | Right parietal | Right posterior temporal | No surgery (SPECT unsuccessful) |
| 12 | Right temporal | Right | Temporal (interictal, ictal) | Temporal, not lateralised | Right temporal | Right mesial/anterior temporal | Right ATLR - gliosis |
| 13 | Right temporal | Right | Posterior temporal (ictal), bitemporal (interictal) | No lateralising/localising | Right temporo-parietal | Right posterior temporal | Right ATLR - MCD type II |
| 14 | Right temporal | Right | Frontotemporal (interictal, ictal) | Bitemporal | Normal | Right anterior temporal | Right ATLR - end folium gliosis |
| 15 | Right temporal | Right | Temporal (interictal, ictal) | Right hemisphere | Right temporal | Right posterior mesial | No surgery (patient declined) |
| 16 | Right temporal | Right | Temporal (interictal, ictal) | Not lateralising/localising | Right temporo-parietal | Right mesial temporal | Right ATLR - end folium gliosis |
| 17 | Right temporal | Right | Temporal (interictal) | Not lateralising/localising | Normal | Right mesial temporal | Right ATLR - end folium gliosis |
| 18 | Temporal, not lateralised | Right | Temporal (interictal, ictal) | No lateralising/localising | Normal | Right mesial temporal | Right ATLR - end folium gliosis |
| 19 | Right temporal | Right | Temporal (ictal) | Right temporal | Right temporal | Right temporal, likely neocortical | Right ATLR - end folium gliosis |
| 20 | Right temporal | Right | Temporal (interictal, ictal) | Right frontotemporal | Right temporal | Right anterior MTG | Right ATLR - FCD IIb |
| 21 | Right temporal | Right | Fronto-temporal right>left (interictal), non-localisable (ictal) | Right temporal | Right temporal | Right temporal (non-localised) | No surgery (awaiting SPECT) |
| 22 | Temporal, not lateralised | Right | Temporal (interictal) | Right temporal | N/A | Right temporal (non-localised) | No surgery (not considered) |

**Supplementary Figure 1** – Difference in T1 signal heterogeneity within the amygdala demonstrated upon the radiological re-review of an MRI negative case.


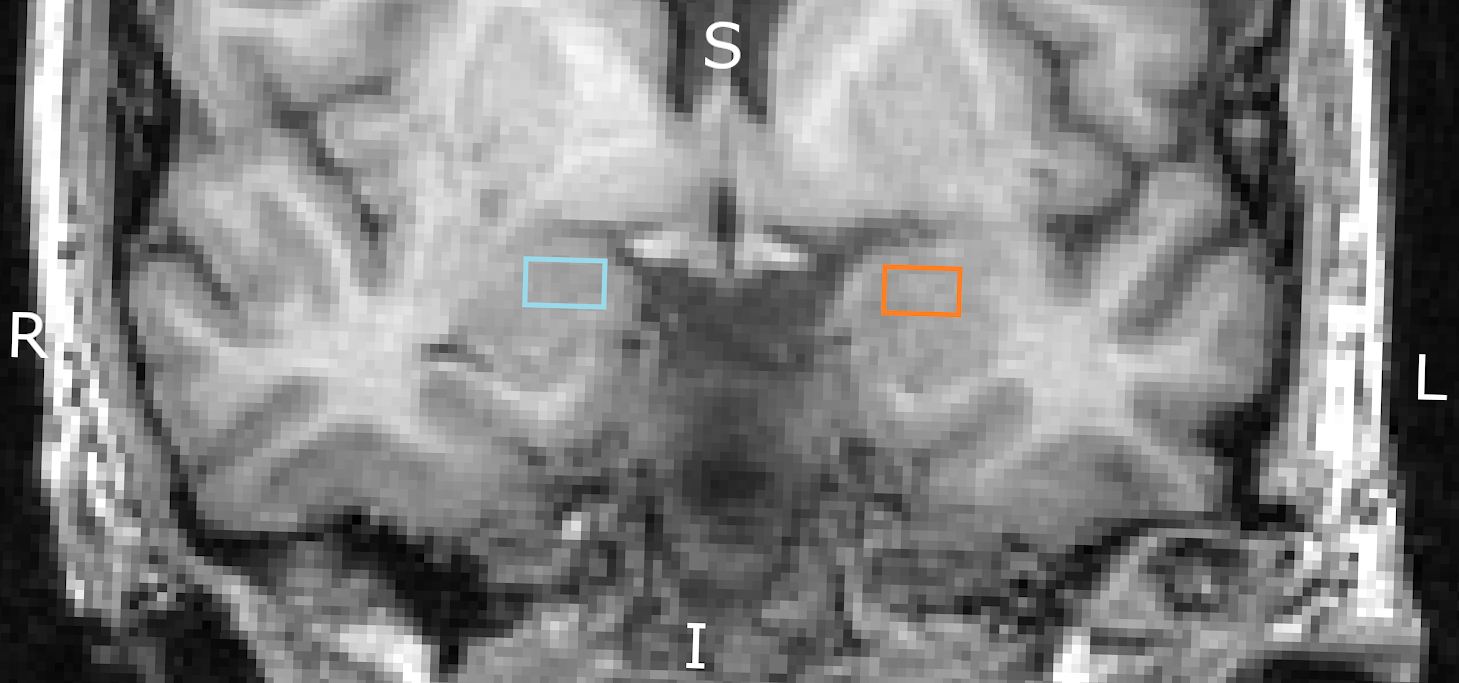


**Supplementary Table 2** – Lateralising imaging features found on radiological re-assessment of the MRI negative cases.

| **Case** | **Lateralising imaging features** |
| --- | --- |
| 1 | None observed |
| 2 | Left amygdala, increased T1 signal heterogeneity |
| 3 | Left inferior temporal gyrus, query small encephalocoele at tegmen tympani |
| 4 | None observed |
| 5 | Left parahippocampal gyrus T1/T2 signal change |
| 6 | None observed |
| 7 | None observed |
| 8 | None observed |
| 9 | None observed |
| 10 | None observed |
| 11 | None observed |
| 12 | None observed |
| 13 | Right hippocampus, increased T2 signal intensity |
| 14 | None observed |
| 15 | Right temporal, increased T1 signal intensity |
| 16 | None observed |
| 17 | Right temporal pole, GM-WM indistinct on T1, and FLAIR |
| 18 | None observed |
| 19 | Right amygdala, mild increase in T2 signal intensity |
| 20 | None observed |
| 21 | None observed |
| 22 | Right amygdala, increased T2/FLAIR signal intensity |
